# Channelling carbon flux through the *meta*‐cleavage route for improved poly(3‐hydroxyalkanoate) production from benzoate and lignin‐based aromatics in *Pseudomonas putida* H

**DOI:** 10.1111/1751-7915.13705

**Published:** 2020-11-10

**Authors:** José Manuel Borrero‐de Acuña, Izabook Gutierrez‐Urrutia, Cristian Hidalgo‐Dumont, Carla Aravena‐Carrasco, Matias Orellana‐Saez, Nestor Palominos‐Gonzalez, Jozef B. J. H. van Duuren, Viktoria Wagner, Lars Gläser, Judith Becker, Michael Kohlstedt, Flavia C. Zacconi, Christoph Wittmann, Ignacio Poblete‐Castro

**Affiliations:** ^1^ Biosystems Engineering Laboratory Center for Bioinformatics and Integrative Biology (CBIB) Faculty of Life Sciences Universidad Andres Bello Santiago Chile; ^2^ Institute of Systems Biotechnology Saarland University Saarbrücken Germany; ^3^ Facultad de Química y de Farmacia Pontificia Universidad Católica de Chile Santiago Chile; ^4^ Institute for Biological and Medical Engineering Schools of Engineering, Medicine and Biological Sciences Pontificia Universidad Católica de Chile Santiago Chile; ^5^ Present address: Institute of Microbiology Technical University of Braunschweig Braunschweig Germany

## Abstract

Lignin‐based aromatics are attractive raw materials to derive medium‐chain length poly(3‐hydroxyalkanoates) (*mcl*‐PHAs), biodegradable polymers of commercial value. So far, this conversion has exclusively used the *ortho‐*cleavage route of *Pseudomonas putida* KT2440, which results in the secretion of toxic intermediates and limited performance. *Pseudomonas putida* H exhibits the *ortho‐* and the *meta‐*cleavage pathways where the latter appears promising because it stoichiometrically yields higher levels of acetyl‐CoA. Here, we created a double‐mutant H‐Δ*catA*Δ*A2* that utilizes the *meta* route exclusively and synthesized 30% more PHA on benzoate than the parental strain but suffered from catechol accumulation. The single deletion of the *catA*2 gene in the H strain provoked a slight attenuation on the enzymatic capacity of the *ortho* route (25%) and activation of the *meta* route by nearly 8‐fold, producing twice as much *mcl*‐PHAs compared to the wild type. Inline, the mutant H‐Δ*catA*2 showed a 2‐fold increase in the intracellular malonyl‐CoA abundance – the main precursor for *mcl‐*PHAs synthesis. As inferred from flux simulation and enzyme activity assays, the superior performance of H‐Δ*catA*2 benefited from reduced flux through the TCA cycle and malic enzyme and diminished by‐product formation. In a benzoate‐based fed‐batch, *P*. *putida* H‐Δ*catA*2 achieved a PHA titre of 6.1 g l^–1^ and a volumetric productivity of 1.8 g l^–1^ day^–1^. Using Kraft lignin hydrolysate as feedstock, the engineered strain formed 1.4 g l^‐ 1^ PHA. The balancing of carbon flux between the parallel catechol‐degrading routes emerges as an important strategy to prevent intermediate accumulation and elevate *mcl‐*PHA production in *P. putida* H and, as shown here, sets the next level to derive this sustainable biopolymer from lignin hydrolysates and aromatics.

## Abstract 2

Engineered *P*.* putida* H sets a benchmark in lignin‐based PHA. Balancing the catechol degrading pathways enables improved PHA production. A DO‐stat fed‐batch process permits the efficient conversion of lignin hydrolysates into biomass and biopolymer.

## Introduction

Microbial polymers are promising alternatives to conventional petrochemical plastics (Choi *et al*., 2019; Becker and Wittmann, 2019, 2016). Particularly attractive biopolymers are poly(3‐hydroxyalkanoates (PHAs), accessible from renewables via microbial conversion and proven biodegradable (Castilho *et al*., 2009; Borrero‐de Acuña *et al*., 2019). More than 160 different monomers can be channelled into PHAs yielding materials with various properties. Most PHAs are thermoplastics, enabling applications as packaging materials, films, drug delivery systems and medical implants (Zhang *et al*., 2018).

In nature, PHAs accumulate as intracellular inclusion bodies in microbial cells under nutrient imbalances (Madison and Huisman, 1999). Among. different bacteria, strains of *P. putida* are metabolically versatile (Poblete‐Castro *et al*., 2019; Weimer *et al*., 2020) and most efficient in accumulating PHAs under nitrogen limitation (Prieto *et al*., 2016; Poblete‐Castro et al., 2020). Previous studies have achieved high PHA production in pseudomonads mostly from sugars (Poblete‐Castro, Rodriguez, *et al*., 2014; Borrero‐de Acuña *et al*., 2014; Davis *et al*., 2015) and sugar alcohols such as glycerol (Poblete‐Castro *et al*., 2014; Beckers *et al*., 2016; Pacheco *et al*., 2019; Orellana‐Saez *et al*., 2019). However, microbes accumulate PHAs also on aromatic substrates (Widdel and Pfennig, 1981; Nikodinovic *et al*., 2008) and in line, PHA production was recently demonstrated from lignin‐based compounds using *P*.* putida* KT2440 (Linger *et al*., 2014; Liu *et al*., 2017) and engineered mutant derivatives (Salvachúa *et al*., 2020). Although the developed *Pseudomonas* strains synthesized only minor PHA levels (0.25 g l^–1^ from lignin‐based aromatics and approximately 1 g l^–1^ from *p*‐coumarate) (Salvachúa *et al*., 2020), these pioneering efforts provide a seminal proof of concept. Lignin displays the second most abundant polymer on earth, is rich in aromatics, widely regarded as a waste and heavily underexploited, thus highly interesting to derive bio‐based chemicals and materials (Dietrich *et al*., 2019; Becker and Wittmann, 2019). Towards future industrialization of lignin‐based PHA, it appears crucial at this point to further enhance microbial production from aromatics and identify the most promising microbial strains and pathways for high PHA yield and titre.

**Fig. 1:**
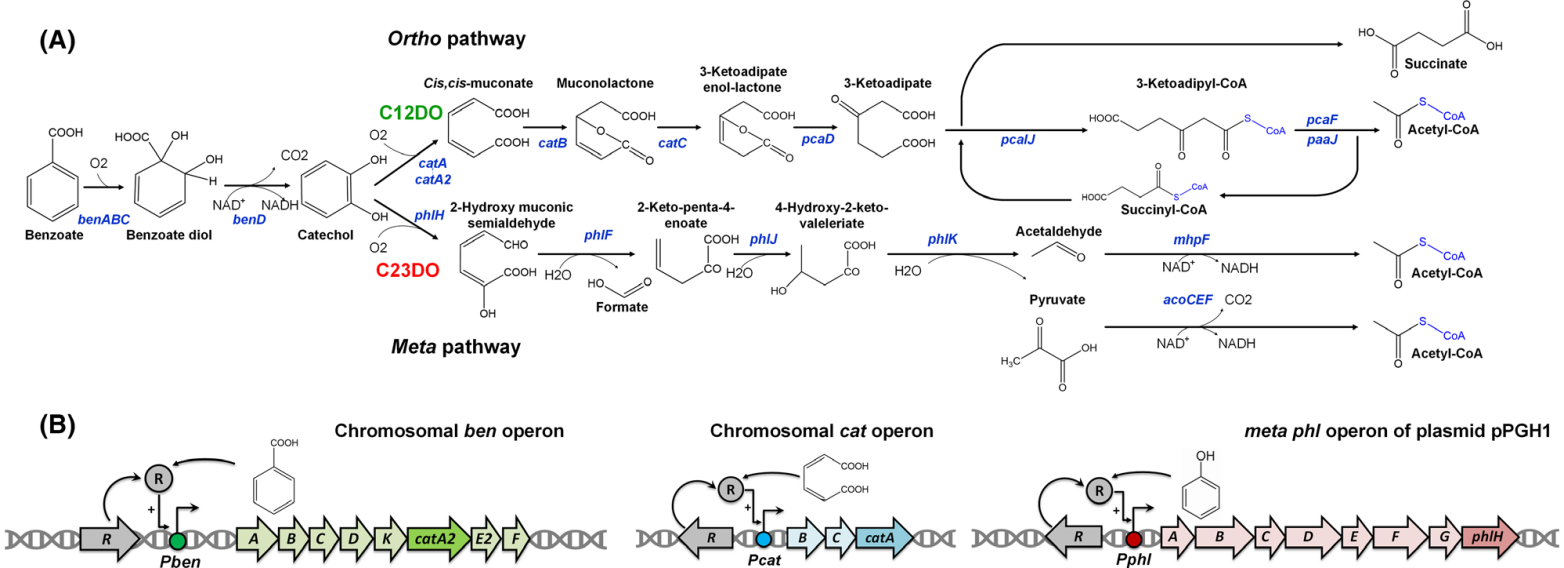
(A) Metabolic degradation of benzoate in *Pseudomonas putida* H through the parallel *ortho*‐ and *meta*‐cleavage pathways yielding different acetyl‐CoAs. B. Genetic organization of the catabolic benzoate (*ben*), catechol (*cat*), phenol (*phl*) operons and their controls in *P. putida* H. Catechol 1,2‐dioxygenase (C12DO) and catechol 2,3‐dioxygenase (C23DO).

Here, we investigated the natural phenol‐degrader *P. putida* H for PHA production. The H strain harbours the plasmid pPGH1 (Herrmann *et al*., 1987), comprising phenol degrading genes encoding for a hydroxylase, metapyrocatechase and 2‐hydroxymuconic semialdehyde dehydrogenase (Herrmann *et al*., 1995) along with the entire genetic machinery for assembling *mcl*‐PHA (Vizoso *et al*., 2015). Strain H displays two parallel routes for benzoate breakdown, downstream of the intermediate catechol (Fig. 1). The *ortho*‐pathway involves catechol 1,2‐dioxygenase (C12DO), yields succinyl‐CoA and acetyl‐CoA and is the exclusive route to degrade benzoate in *P. putida* KT2440 (Jiménez *et al*., 2002). In addition, *P. putida* H operates the *meta* pathway which involves catechol 2,3‐dioxygenase (C23DO) and forms pyruvate and acetyl‐CoA, and considering downstream action of pyruvate dehydrogenase, ultimately forms 2 acetyl‐CoA (Fig. 1A). Our knowledge regarding the contribution of the two pathways during PHA accumulation, i.e. nitrogen limitation, is rather poor. Notably, acetyl‐CoA is the starter unit to derive malonyl‐CoA, the main precursor for the synthesis of PHAs. In our study, a set of *P. putida* H mutants with engineered aromatic catabolism was generated and characterized in detail. The best strain *P. putida* H‐Δ*catA2* used simultaneously both the *ortho* and *meta* routes, achieved a PHA titre of 6.3 (g l^–1^) from benzoate in a fed‐batch process and surpassed previous PHA production efforts on aromatic compounds approximately six‐fold.

## Results

### Genomic repertoire of *P. putida* H to produce PHA from aromatics and construction of ortho‐cleavage pathway mutants

As inferred from the genome analysis, *P. putida* H harbours two chromosomal genes that encode for catechol‐1,2‐dioxygenases (C12DO) in the *ortho*‐cleavage pathway for the breakdown of benzoate. One gene was in the *cat* operon and the other one belonged to the *ben* operon (Fig. 1B). The alignment of amino acid sequence of the *ortho*‐pathway enzymes CatA and CatA2 of strain H against the homologues of *P. putida* KT2440 (Jiménez *et al*., 2002) showed 100% identity. We therefore designated them *catA* and *catA2*, as previously done for KT2440 (van Duuren *et al*., 2011). Besides, and different to KT2440, *P. putida* H harbours the plasmid‐based gene *phlH*, encoding a catechol‐2,3‐dioxygenase (C23DO) as the entry step of the meta pathway (Fig. 1). Different deletion strains were constructed to (i) partially and (ii) completely block the *ortho* route for aromatic conversion. The obtained mutants *P. putida* H‐Δ*catA,* H‐Δ*catA2,* H‐Δ*catA*Δ*A2*, lacking either one or both C12DOs were validated by PCR. The H‐Δ*catA* deletion yielded a shortened fragment (1173 bp) as compared to wild type (2019 bp), when using the oligos catAUpFw/DwRv. Likewise, the fragment size for Δ*catA2* (1095 bp) was smaller than in wild type (2010 bp), using the catA2UpFw/DwRv primers. Positive clones were additionally verified by sequencing.

### Growth and PHA synthesis of *P. putida* H ortho‐pathway mutants on benzoate

**Fig. 2:**
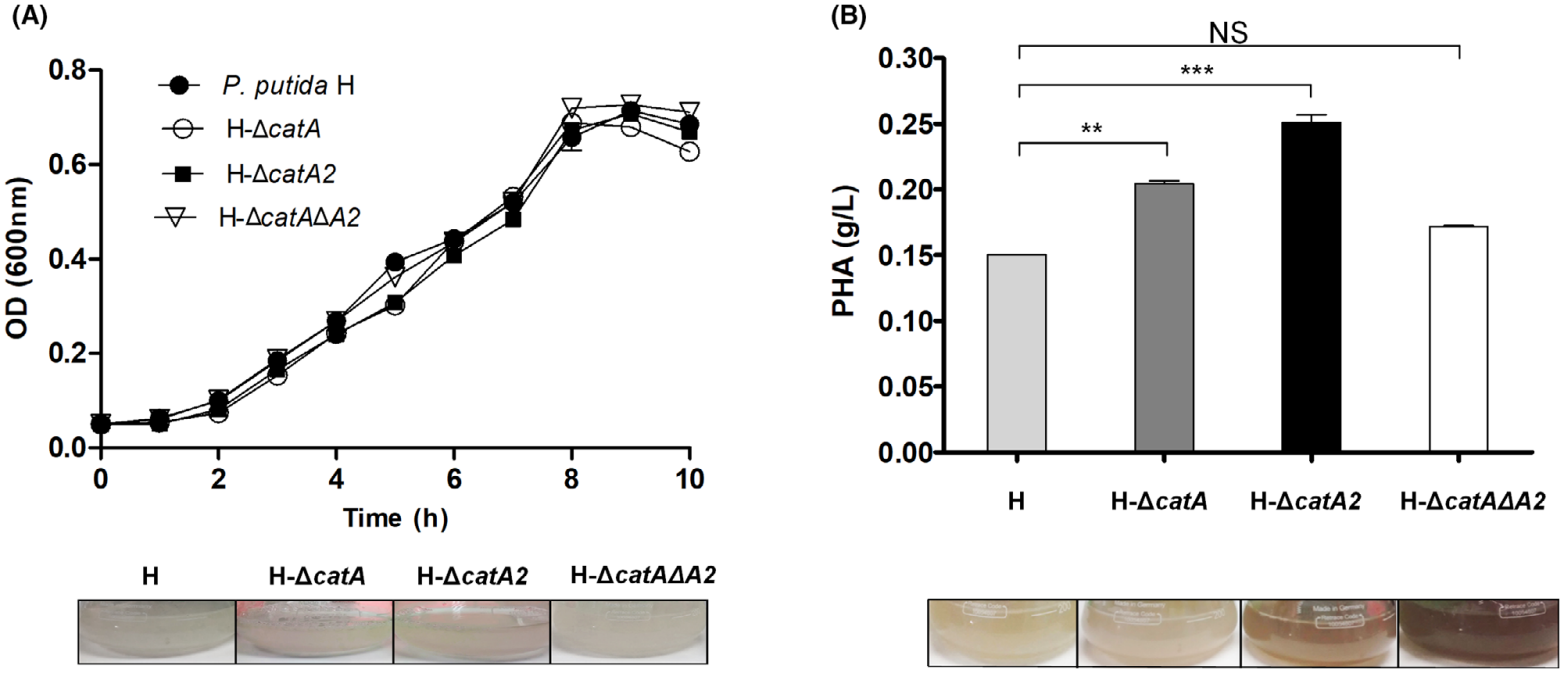
A. Growth of *P. putida* H and its catabolic pathway mutants on 5 mM benzoate (C/N ratio 0.3 mol mol^–1^). B. PHA production by the engineered *P. putida* H strain after 24 h cultivation (C/N ratio 4.0 mol mol^–1^). Values represent the mean and errors from three independent experiments. NS, not significant. *P* values (*P*> 0.05, *P < 0.05, **P < 0.001 and ***P < 0.0001).

First, the growth of the single‐deletion mutants (H‐Δ*catA* and H‐Δ*catA2*), the double deletion mutant (H‐Δ*catA*Δ*A2*), and the parental strain H was characterized on benzoate with sufficient ammonium supply (C/N ratio 0.3 mol mol^–1^) (Fig. 2A). All mutants retained the maximum specific growth rate of *P. putida* H (Table 1). In a previous work of benzoate‐grown *P. putida* KT2440, carrying the TOL plasmid pWW0, inactivation of *catA*2 promoted the secretion of 2‐hydroxymuconic semialdehyde (2‐HMS), an intermediate of the *meta* route resulting in a yellowish culture broth (Jiménez et al., 2014). We did not observe such colour in any culture, including that of the corresponding H‐Δ*catA2* mutant (Fig. 2A).

**Table 1:** Physiological parameters of *P. putida* H and metabolically engineered mutants grown on 5 mM benzoate and 1 g l^–1^ NH_4_Cl in shake flasks. Values represent the mean and corresponding errors from three independent experiments.

Strain	Specific growth rate (h^–1^)	Biomass yield (g_CDW_ mol^–1^)	Specific benzoate uptake rate (mmol g_CDW_^–1^ h^–1^)
*P. putida* H	0.33 ± 0.01	67.16 ± 2.44	4.91 ± 0.04
H‐Δ*catA*	0.35 ± 0.00	72.05 ± 1.22	4.86 ± 0.06
HΔ*catA*2	0.34 ± 0.01	70.82 ± 2.44	4.80 ± 0.03
H‐Δ*catA*Δ*A*2	0.33 ± 0.02	72.05 ± 1.22	4.52 ± 0.01

We then assessed the PHA production capacity of all strains by growing them on an increased ratio of benzoate to ammonium (final C/N ratio 4.0 mol mol^–1^) and quantified the PHA amount at 24 h cultivation in batch cultures. As shown in Fig. 2B, wild‐type *P. putida* H produced 0.14 g l^–1^ PHA under these conditions. The inactivation of the *catA* gene increased the amount of PHA by 50% to 0.21 g l^–1^. The deletion of *catA*2 boosted PHA synthesis even stronger to 0.24 g l^–1^, 70% more than the parental H strain. The double‐mutant H‐Δ*catA*Δ*A*2, however, presented an almost equal PHA level as compared to the wild type. In this case, the culture broth turned dark brownish, an indication of catechol accumulation and its oxidation and polymerization during the bioconversion process (Jiménez et al., 2014) (Fig. 2B). A slightly visible brown colour was observed for the best producer H‐Δ*catA2*, whereas the cultures of wild‐type and strain H‐Δ*catA* remained clear.

**Fig. 3:**
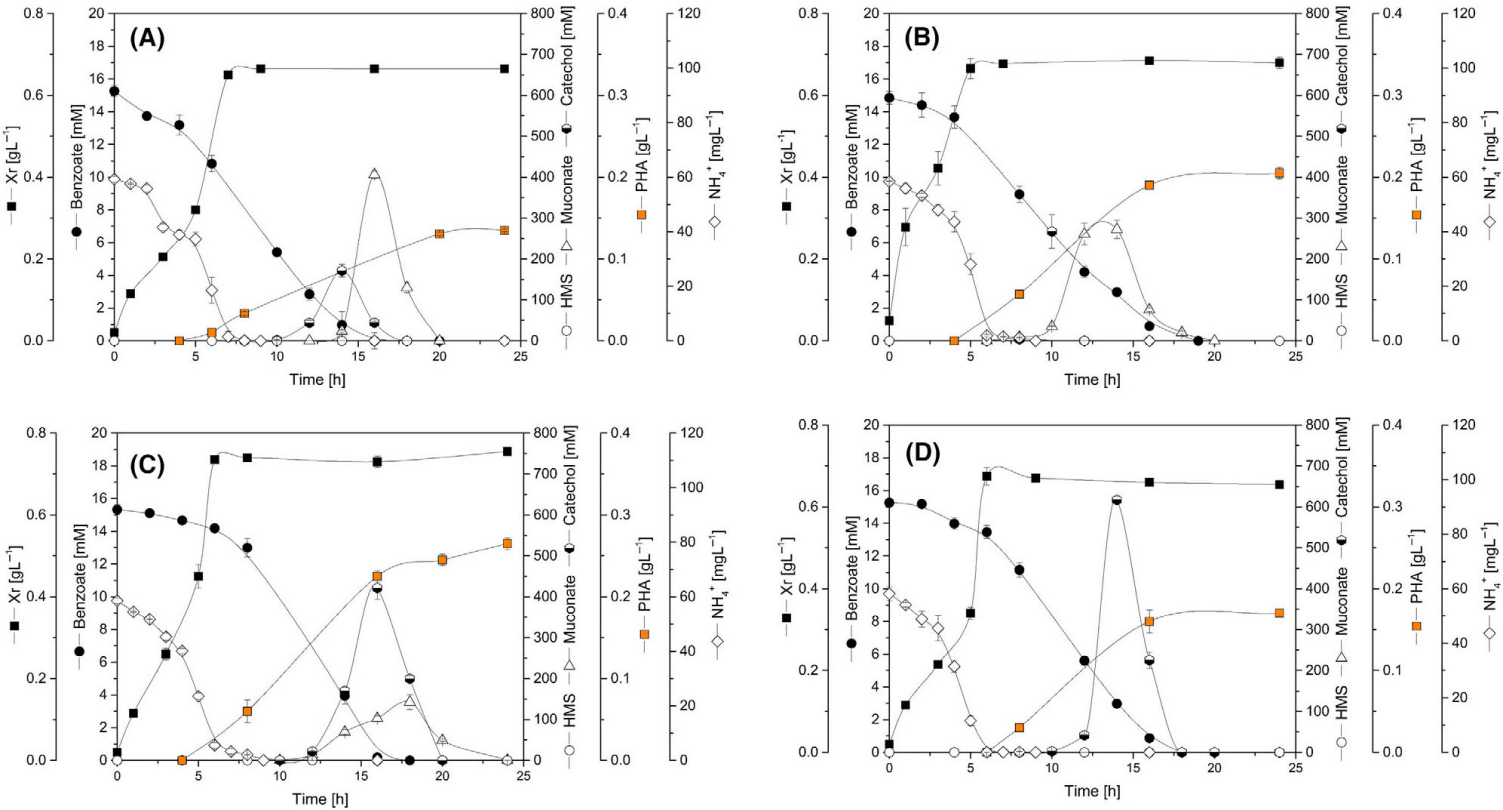
Time course of benzoate degradation and biomass, catechol, muconate, 2‐HMS and PHA synthesis by *P. putida* H strains (A) wild‐type, (B) H‐Δ*catA*, (C) H‐Δ*catA*2, (D) H‐Δ*catA*Δ*A*2 in batch bioreactors. (C/N) ratio 4 (mol mol^–1^). Values represent the mean and errors from two independent bioreactors.

Next, we performed cultivations in bioreactors to study growth and production dynamics in more detail. Using the previous high benzoate to ammonium ratio, nitrogen limitation triggered PHA accumulation in later phases of the process (Fig. 3, Table 2). The single‐deletion strains H‐Δ*catA* and H‐Δ*catA*2 accumulated the highest level of PHA, 0.20 and 0.26 g l^–1^ after 24 h cultivation respectively. During cultivation, they secreted and then re‐consumed catechol and *cis,cis*‐muconate. The latter metabolite exclusively occurs as intermediate of the *ortho* route (Fig. 1), indicating that this branch was involved in catechol conversion in both strains. The strain H‐Δ*catA*2 revealed a higher catechol peak, correlating to a more pronounced culture pigmentation, than the wild‐type and strain H‐Δ*catA*. Interestingly, the two intermediates peaked later in H‐Δ*catA*2 than in the other two strains. None of the strains accumulated 2‐HMS, suggesting a sufficient bioconversion capacity of the *meta* route (Fig. 3B‐C). The H‐Δ*catA*2 mutant achieved the highest PHA level among all strains (Table 2). The double mutant did not accumulate *cis,cis*‐muconate but secreted substantial amounts of catechol. It reached a PHA titre of 0.17 g l^–1^, 30% more than the wild type, but significantly less than the single‐deletion strains (Table 2).

**Table 2:** Biomass synthesis, PHA concentration and monomer composition of wild‐type *P. putida* H and metabolically engineered strains in batch cultures using top‐bench bioreactors on 15 mM of sodium benzoate. Values represent the mean and corresponding errors from two independent experiments.

Strain	CDW (g l^–1^)	PHA (g l^–1^)	PHA (%wt)	Monomer composition (%)^a^			
				C8	C10	C12	C12:1
*P. putida* H	0.79 ± 0.01	0.13 ± 0.00	16.5 ± 0.1	11.8 ± 0.4	81.2 ± 1.3	6.3 ± 0.4	0.7 ± 0.1
H‐Δ*catA*	0.87 ± 0.06	0.20 ± 0.01	23.0 ± 0.4	9.7 ± 0.5	82.1 ± 1.8	6.8 ± 1.8	1.5 ± 0.1
H‐Δ*catA*2	1.00 ± 0.05	0.26 ± 0.01	26.0 ± 0.8	14.3 ± 1.1	75.3 ± 2.2	3.9 ± 0.1	6.5 ± 0.3
H‐Δ*catA*Δ*A*2	0.86 ± 0.02	0.17 ± 0.00	19.8 ± 0.3	7.9 ± 0.8	75.6 ± 1.6	8.4 ± 0.3	8.1 ± 0.1

^a^ C8: 3‐hydroxyoctanoate, C10: 3‐hydroxydecanoate, C12: 3‐hydroxydodecanoate, C12:1: 3‐hydroxy‐5‐cis‐dodecanoate.

All mutants showed an interesting variation in PHA monomer composition. Whereas 3‐hydroxydecanoate (C10) generally was the major monomer (75–80%) (Table 2), all mutants revealed an increased content of carbon‐twelve building blocks as compared to the wild type. This effect was most pronounced for the double mutant, which exhibited nearly a two‐fold increase of C12:0 and C12:1 (16.5%) as compared to strain H (7.0%), while H‐Δ*catA* (8.3%) and H‐Δ*catA*2 (10.4%) showed values in between.

### Intracellular levels of CoA thioesters in wild‐type and engineered *P. putida* H during growth and PHA production

**Fig. 4:**
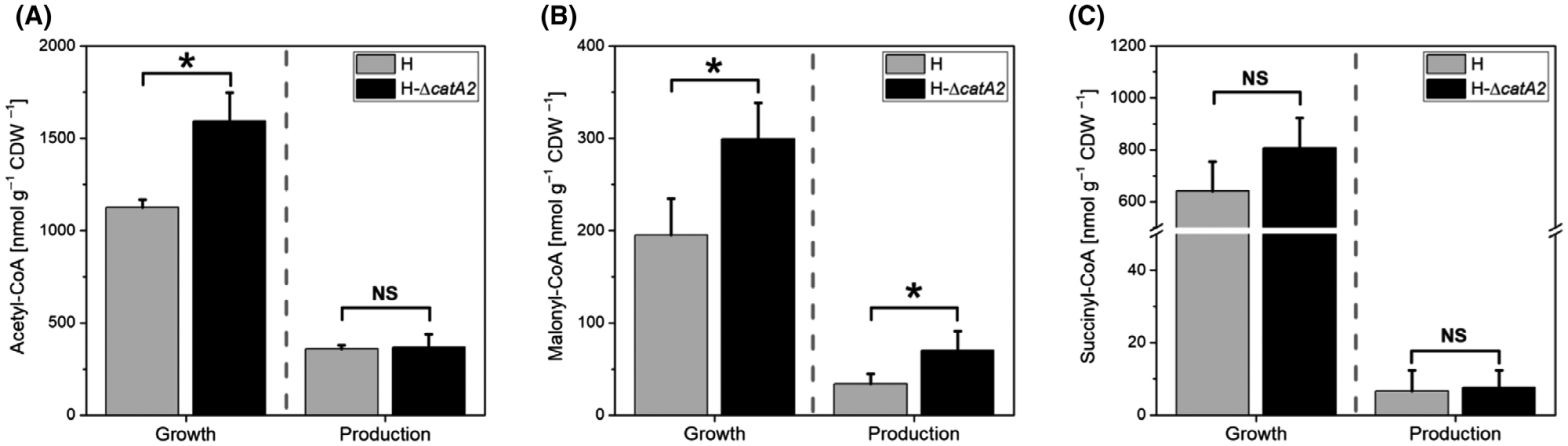
Intracellular level of CoA thioesters in *P. putida* H and its *catA*2 deletion mutant during growth (sample taken at 5 h cultivation under nitrogen excess) and PHA production (sample taken at 15 h cultivation under nitrogen limitation) in batch cultures. A. Acetyl‐CoA levels (nmol gCDW^–1^), (B) malonyl‐CoA levels (nmol gCDW^–1^), (C) succinyl‐CoA levels (nmol gCDW^–1^). Values represent the mean and errors from three independent experiments. NS, not significant. *P* values (*P*> 0.05 and *P < 0.05).

The imposed growth conditions in the batch cultures revealed a two‐phase profile when grown on a high benzoate to ammonium ratio: initial growth if sufficient nitrogen was available and subsequent PHA accumulation under nitrogen limitation (Fig. 3). We thus quantified the intracellular CoA thioesters, including the PHA precursor malonyl‐CoA and its upstream intermediate acetyl‐CoA (Fig. 4). Samples were taken from the wild‐type and the best PHA producer H‐Δ*catA*2 during growth (5 h cultivation, nitrogen excess) and PHA synthesis (15 h cultivation, nitrogen limitation) in batch cultures. During growth, the mutant H‐Δ*catA*2 – expressing the attenuated *ortho*‐pathway – exhibited 50% increased acetyl‐CoA and malonyl‐CoA levels as compared to the wild type, whereas the TCA cycle intermediate succinyl‐CoA was not affected. Notably, the concentration of the CoA esters generally collapsed when cells entered nitrogen limitation. For example, the availability of succinyl‐CoA was decreased up to a hundred‐fold. Regarding PHA production, the mutant H‐Δ*catA*2 exhibited twice as much malonyl‐CoA during the production phase (Fig. 4B).

### Kinetics of PHA synthesis in chemostat cultures

To obtain rates and yields undermaintained nitrogen‐limiting conditions and provide the experimental basis for later steady‐state flux analysis (see below), specific benzoate uptake and production rates (biomass, PHA, carbon dioxide and by‐products) were assessed in chemostat cultures, operated under benzoate excess and nitrogen‐limiting conditions (C/N ratio of 4.02 mol mol^–1^) to promote PHA accumulation (Table 3). Once cultures had reached a steady state, the measurements yielded a well‐closed carbon balance for all strains, underlining the data’s consistency. The wild type produced PHA at 21% on a cell dry basis. Almost 50% of the benzoate taken up accumulated as *cis,cis*‐muconic acid. In comparison, the mutant strain H‐Δ*catA* revealed an increased PHA content (27%). Here, catechol was secreted together with *cis,cis*‐muconic acid. As observed in batch cultures, *P. putida* H‐Δ*catA*2 exhibited the best performance. It consumed benzoate fastest and exhibited the highest PHA titre and content (35%), more than twice as high as the wild type. Notably, the two deletion strains produced significantly more CO_2_. This effect might have resulted from the usage attenuation of the *ortho*‐pathway in the two mutants, which could have otherwise channelled more aromatics to succinyl‐CoA downstream of the dehydrogenases, the entry of the TCA cycle like pyruvate dehydrogenase (PDH), 2‐oxoglutarate dehydrogenase (ODH) and isocitrate dehydrogenase (ICDH). The double knockout mutant strain, which exclusively converted catechol via the *meta* pathway, obviously suffered from the high level of secreted catechol. It showed rather low rates regarding substrate uptake, PHA formation and CO_2_ evolution.

**Table 3:** Growth kinetics and stoichiometry of *P. putida* strains grown on 15 mM benzoate under nitrogen‐limiting conditions in chemostat cultures. D = 0.09 (h^–1^). Values represent the mean and corresponding errors from two independent experiments.

Strain	Residual benzoate (mmol)	Y_Xr/S_ (g mol^–1^)	Y_PHA/S_ (g mol^–1^)	PHA^a^ (wt%)	Carbon recovery (%)	Uptake and production rates (mmol gCDW^–1^ h^–1^)					
						Benzoate	*Cis,cis*‐muconate		Catechol	CO_2_	
*P. putida* H	9.4 ± 0.6	44.6	11.5	21.1	97 ± 3	2.39 ± 0.17		1.15 ± 0.09	0.02 ± 0.17		4.19 ± 0.31
H‐Δ*catA*	7.7 ± 0.4	43.2	15.9	27.0	95 ± 2	2.16 ± 0.12		0.42 ± 0.01	0.34 ± 0.02		5.51 ± 0.28
H‐Δ*catA*2	7.3 ± 0.5	47.6	24.5	35.4	102 ± 3	2.64 ± 0.08		0.93 ± 0.06	0.12 ± 0.03		6.72 ± 0.45
H‐Δ*catA*Δ*A*2	6.4 ± 0.8	69.2	11.6	15.0	96 ± 6	1.54 ± 0.14		0.00	0.52 ± 0.02		3.89 ± 0.10

^a^ PHA content relative to cell dry weight (CDW).

### Impact of the inactivation of catA genes on catechol‐dioxygenase activities under PHA synthesis

**Fig. 5:**
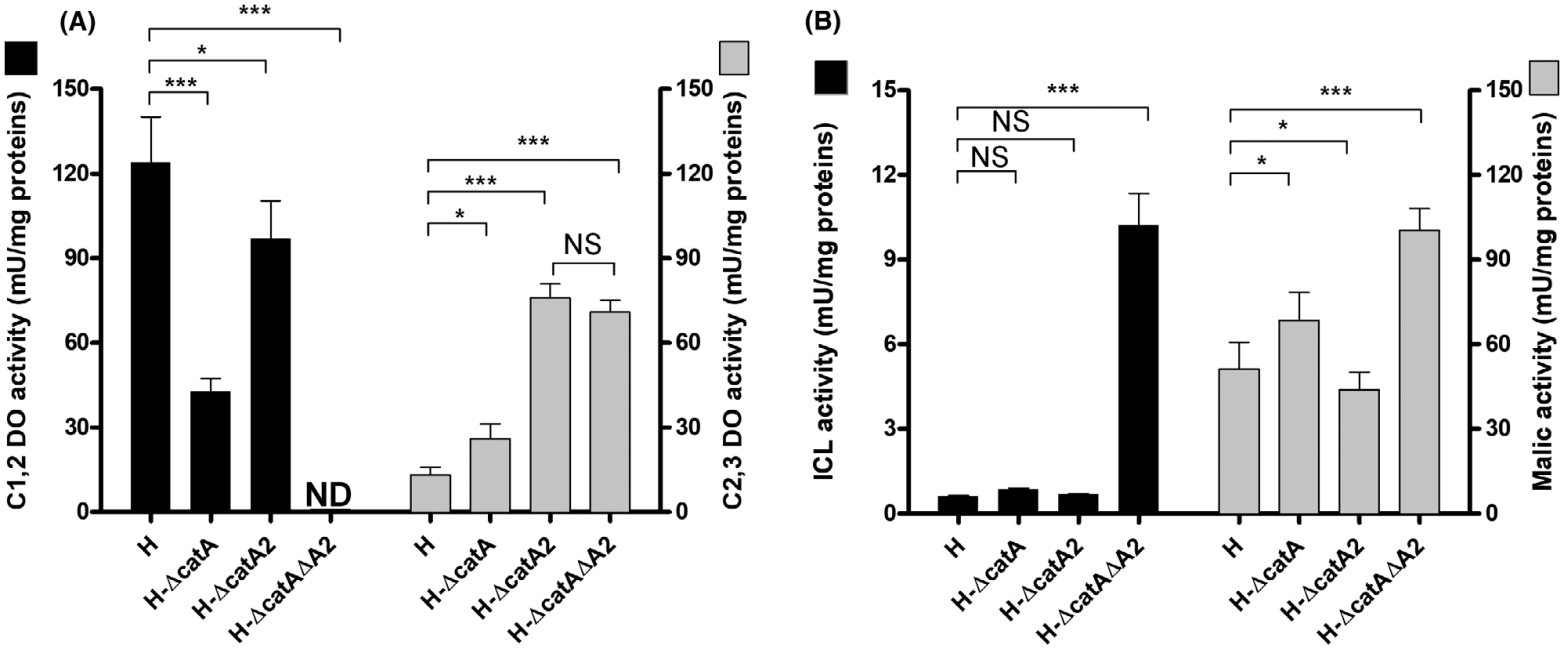
A. Activities of catechol 1,2‐dioxygenase (C12DO) and catechol 2,3‐dioxygenase (C23DO). B. Activities of isocitrate lyase (ICL) and malic enzyme (MAE) in *P. putida* H and catabolic pathway mutants, grown in chemostat (D = 0.09 h^–1^) on benzoate under PHA‐producing conditions. Values represent the mean and errors from three independent experiments. ND, not detected. (NS, not significant (*P*> 0.05), *P < 0.05, **P < 0.001 and ***P < 0.0001).

As shown, the different mutants varied significantly regarding growth and PHA production and differed in the extent of intermediate catabolic secretion. The capacity of the *ortho* and the *meta* route downstream of the central node catechol was now studied by measuring the capacity of the catechol dehydrogenases (C12DO and C23DO) from the chemostat cultures. The wild‐type *P. putida* H expressed enzymes of both routes (Fig. 5A). The activity of C12DO, reflecting the two *ortho* cleaving dioxygenases, was almost ten‐fold higher than that of C23DO, the enzyme of the *meta* route, suggesting the *ortho* branch as dominating catabolic pathway. The inactivation of *catA* reduced the C12DO activity to 30%, whereas approximately 75% C12DO activity was retained in H‐Δ*catA*2, indicating a substantially different impact of the two enzymes on pathway flux. Surprisingly, deletion of either *catA* or *catA2* had a strong effect on the enzymatic capacity of the *meta* route. While inactivation of *catA* provoked a two‐fold enhanced activity of C23DO, *catA2* deletion resulted in an eight‐fold activity increase for C23DO to 75 (mU mg^–1^), the highest activity among the tested strains (Fig. 5A).

Taken together, *P. putida* H‐Δ*catA*2 exhibited the highest total capacity to metabolize catechol (180 mU mg^–1^), more than 30% higher than in wild type. The double mutation revealed a similarly boost of the C23DO activity, but had lost entirely the C12DO activity so that its total capacity to convert catechol was only 50% of that of the parent and below 40% of that of the best PHA producer *P. putida* H‐Δ*catA*2, explaining its generally reduced performance (Table 3).

### Impact of the catabolic operation between the ortho and meta route on the activity of anaplerotic enzymes

We further measured the activity of two enzymes competing with PHA synthesis for acetyl‐CoA, i.e. the glyoxylate shunt enzyme isocitrate lyase (ICL) and malic enzyme (malate dehydrogenase, MAE), converting the product of the shunt (malate) into pyruvate (Klinke *et al*., 2000)(Borrero‐de Acuña *et al*., 2014)(Fig. 5B). We recorded different ICL activity (0.3–0.6 mU mg ^−1^) in the wild‐type and the two single‐deletion strains H‐Δ*catA* and H‐Δ*catA*2. Likewise, MAE was different between in the strains, whereby the specific activity (5 ‐ 7 mU mg^−1^) was up to 25‐fold higher than that of ICL. Both enzymes were strongly activated in the strain H‐Δ*catA*Δ*A2* (Fig. 5). This activation suggested a partial redistribution of carbon towards the glyoxylate shunt and subsequent decarboxylation of malate into pyruvate via MAE.

### Metabolic fluxes of the engineered PHA‐producing strains

**Fig. 6:**
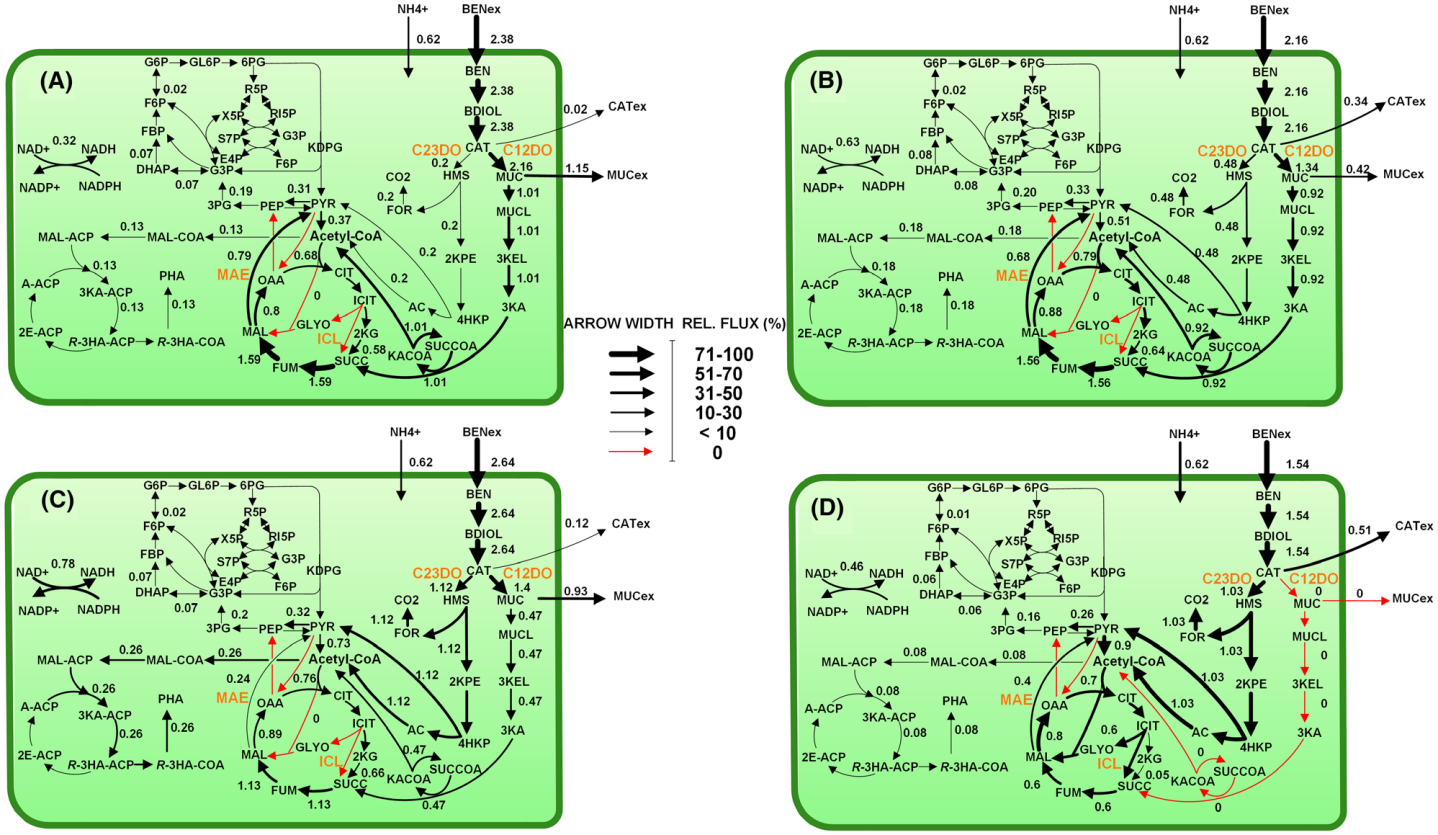
Carbon flux distribution of (A) *P. putida* H, (B) the deletion mutants H‐Δ*catA*, (C) H‐Δ*catA*2 and (D) H‐Δ*catA*Δ*A*2. The rates, used to calculate the fluxes, were inferred from chemostat cultures (D = 0.09 h^–1^) on benzoate under nitrogen‐limiting PHA‐producing conditions. The enzymatic activities for Catechol 1,2‐dioxygenase (C12DO), catechol 2,3‐dioxygenase (C23DO), malic dehydrogenase (MAE), isocitrate lyase (ICL) were measured experimentally. Red arrows represent zero flux.

We used the measured rates (benzoate, catechol, *cis,cis*‐muconate, CO_2_ and PHA, Table 3) and specific dioxygenase activities at the catechol node (Fig. 5) as constrains for metabolic flux balance analysis (FBA) of each strain under PHA‐producing conditions. This procedure allows us to optimize biomass synthesis (FBA) and compare the computed *insilico* growth rate with the imposed specific growth rate (dilution rate *D*) in the chemostats, where the values were very consistent in all cases (0.09 (h^–1^), Fig. 6). At the catechol node, *ortho* and *meta* pathway fluxes were partitioned according to the available enzymatic capacity as previously described (Kind *et al*., 2014; Becker *et al*., 2018)(Fig. 5A, Fig. 6). The enzyme capacity in the wild type comprised that of C23DO (13.3 mU mg protein^–1^) and C12DO (124.0 mU mg protein^–1^). Considering a cellular protein content of 50% (van Duuren *et al*., 2013), this capacity would allow for a maximum flux of 1.6 mmol g_CDW_^–1^ h^–1^ (C23DO) and 14.4 mmol g_CDW_^–1^ h^–1^, (C12DO) respectively (˜1:9 ratio). When both values were added together, they exceeded the measured uptake rate of sodium benzoate, indicating that the catabolic pathway operated below the highest possible flux (Fig. 6) When computing the remaining fluxes, the wild type revealed an active TCA cycle with a citrate synthase flux (at the entry into the cycle) of 0.68 mmol g_CDW_^–1^ h^–1^. The glyoxylate shunt carried nearly zero flux along with an inactive Entner–Doudoroff (ED) pathway (Fig. 6A). The calculation further revealed that the malic enzyme was active.

When comparing the flux pattern of *P. putida* H to that of the single‐deletion mutant H‐Δ*catA*, MAE exhibited a similar flux (Fig. 6A, B). For the mutant H‐Δ*catA2*, however, a lower flux was estimated (Fig. 6C). As more PHA was synthesized by H‐Δ*catA*2, this was reflected by a higher PHA flux 2‐fold than that of the parental strain. A different scenario was found for the double‐mutant H‐Δ*catA*Δ*A*2, where benzoate was only metabolized through the *meta* route. The C23DO enzyme seemed to be less effective at converting catechol to HMS so that catechol secretion was boosted more than 20‐fold (Fig. 6D). There was also a major flux change observed within the TCA cycle, where the flux through the succinic dehydrogenase enzyme was reduced by 50% and the glyoxylate shunt was found activated.

Next, we explored the carbon partitioning between the *meta* and *ortho*‐pathway in more detail. For this, we performed a robustness analysis of fluxes taking the PHA synthesis on benzoate as objective (Edwards and Palsson, 2000). The simulations showed that a redirection of flux exclusively via the *ortho* route (Fig. S1A) resulted in diminished PHA biosynthesis with an undesired high secretion of *cis,cis*‐muconate (Fig. S1B). On the other hand, exclusive use of the *meta* pathway for benzoate conversion led to an enhanced but restricted PHA production flux due to the limited carbon conversion capacity of the C23DO (1.1 mmol g_CDW_^–1 ^h^–1^) under nitrogen limitation (Table 3, Fig. 6D), ultimately leading to the high accumulation of catechol during benzoate transformation (Table 3). These results suggest that a balanced operation of the *meta* and *ortho* route is crucial for an appropriate distribution of catechol via the parallel pathways (Fig. 6, Fig. S1) to enable fast substrate uptake and enhanced formation of acetyl‐CoA for increased PHA synthesis, as observed in the H‐Δ*catA*2 mutant strain.

### *Benchmarking PHA production in* P. putida *H‐ΔcatA2 using a benzoate‐based fed‐batch process*

**Fig. 7:**
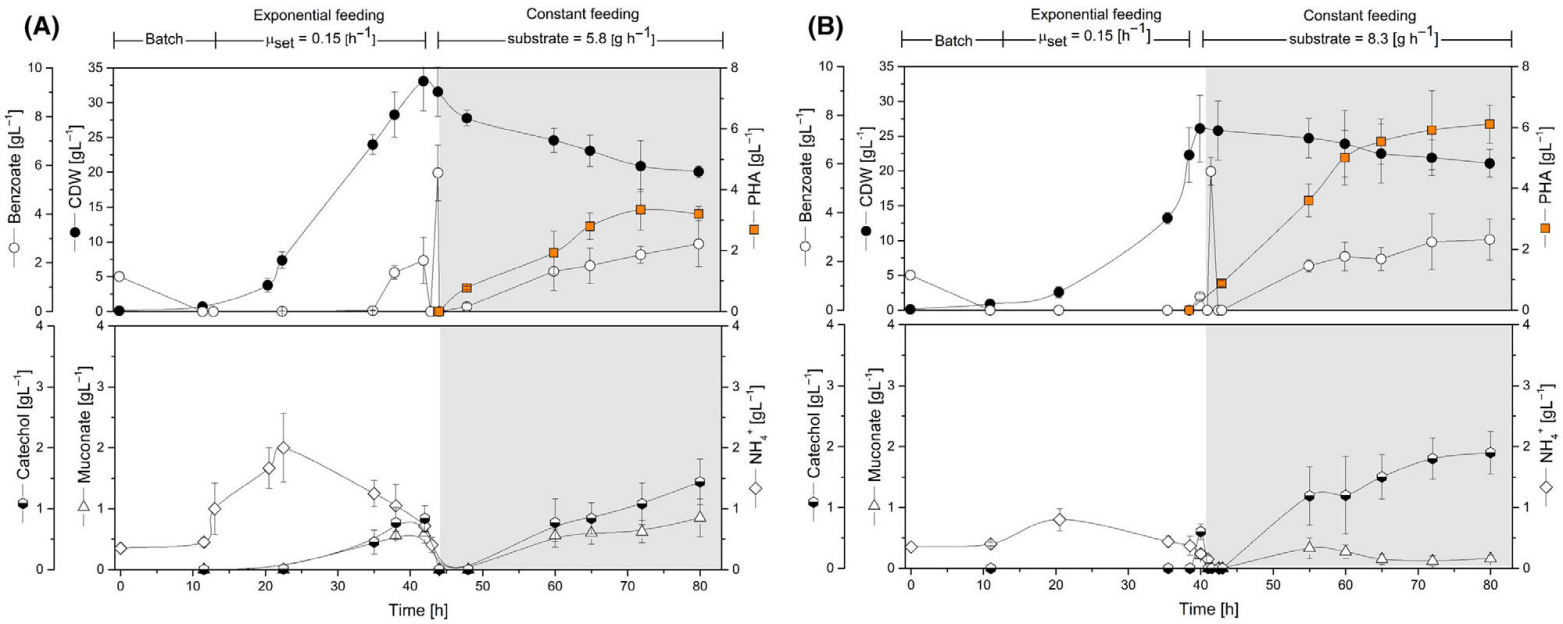
Three‐phase fed‐batch PHA synthesis by feeding benzoate as the solely carbon substrate. The fermentation comprised multiple feeding stages: (i) batch culture (initial benzoate concentration 10 mM), (ii) exponential feeding (µ_set_ 0.15 h^–1^), (iii) constant feeding of benzoate. A. Substrates and products profile of *P. putida* H and (B) the mutant H‐Δ*catA*2. Grey panels indicate nitrogen limitation. Values represent the mean and errors from two independent bioreactors.

The best mutant was now compared to the wild type for PHA production performance using fed‐batch fermentation (Fig. 7). A specific feed strategy was elaborated for this purpose, which comprised three phases. As exemplified for the wild type, an initial batch phase (0–10 h) was followed by exponential feeding of benzoate under sufficient nitrogen availability (ammonium feeding rate 0.11 g h^–1^) to form biomass (10–40 h). This strategy allowed to avoid the accumulation of intermediates, e.g. catechol and *cis,cis*‐muconate. As displayed in Fig. 7A *. P. putida* H reached 34 g_CDW_ l^–1^ after 40 h cultivation. Slightly afterwards, a third phase was initiated. The supply of ammonium was stopped, and a pulse of benzoate was added to deplete the remaining ammonium and trigger PHA accumulation. Once benzoate and ammonium were depleted, the carbon substrate was fed to the bioreactor at a constant rate of 5.8 (g benzoate h^–1^) based on data from the batch cultures during the nitrogen‐limiting phase (Fig. 3). Benzoate slightly accumulated during the final phase, and catechol and *cis,cis‐*muconate were inevitably synthesized to a small extent, hence we tried to maintain the benzoate level as low as possible with the imposed conditions. The cells finally produced 3.3 g l^–1^ of PHA, 16.5% of biomass, at a volumetric productivity of 1.1 g l^–1^ day^–1^.

Using the same feeding approach than for the wild‐type strain during the exponential growth phase (NH_4_Cl feeding rate 0.11 g h^–1^), the mutant H‐Δ*catA2* achieved a slightly lower biomass concentration (27 g l^–1^) after 40 h cultivation (Fig 7B). During the PHA production phase, we stopped the addition of ammonium and supplied benzoate to the bioreactor at a rate of 8.3 g h^–1^, as imposed from the batch behaviour of the mutant (Fig. 3). The engineered H‐Δ*catA2* strain secreted less *cis,cis*‐muconate and slightly higher amounts of catechol (Fig. 7A, B). Notably, the strain synthesized 6.1 g l^–1^ of PHA, resulting in a volumetric productivity of 1.83 g l^–1^ day^–1^, nearly two‐fold higher than the parental strain (Fig. 7B, Table 4).

**Table 4:** Performance of *P. putida* strains for the synthesis of *mcl*‐PHA from benzoate and lignin‐based aromatics

Strain	Fermentation mode	Substrate	CDW (g l^–1^)	PHA (g l^–1^)	PHA productivity (g l^–1^ day^–1^)	Monomer composition (%)^a^						References
						C6	C8	C10	C12	C12:1	C14	
*P. putida* H	Fed‐batch	Benzoate	20.5 ± 1.2	3.3 ± 0.3	1.1 ± 0.1	ND	19.6	77.4	2.3	0.7	ND	This study
H‐Δ*catA*2	Fed‐batch	Benzoate	21.1 ± 0.9	6.1 ± 0.6	1.8 ± 0.2	ND	13.5	78.3	6.8	1.5	ND	This study
KT2440	Batch	Benzoate	0.3	0.1	0.1	6.3	52.0	28.1	4.8	8.5	ND	Xu *et al*. (2019)
H‐Δ*catA*2	Fed‐batch	Enriched lignin stream^b^	5.4 ± 0.3	1.4 ± 0.1	0.7 ± 0.0	ND	11.8	79.4	7.6	1.2	ND	This study
AG2162	Batch	Lignin‐ aromatics	0.7	0.1	0.03	ND	10.0	80.0	8.3	ND	1.7	Salvachúa *et al*. (2020)
KT2440	Fed‐batch	Lignin‐aromatics	5.1	1.0	0.6	NS	NS	NS	NS	NS	NS	Liu *et al*. (2017)
KT2440	Batch	Lignin‐ aromatics	0.8	0.3	0.1	ND	22.0	55.0	16.0	ND	7.0	Linger *et al*. (2014)

ND, Not detected; NS, Not shown.

^a^ C6: 3‐hydroxyhexanoate, C8: 3‐hydroxyoctanoate, C10: 3‐hydroxydecanoate, C12: 3‐hydroxydodecanoate, C12:1: 3‐hydroxy‐5‐*cis*‐dodecanoate.

^b^ The feeding solution was enriched with pure catechol (final concentration 250 mM catechol).

### Fed‐batch PHA production on lignin‐derivative aromatic compounds

**Fig. 8:**
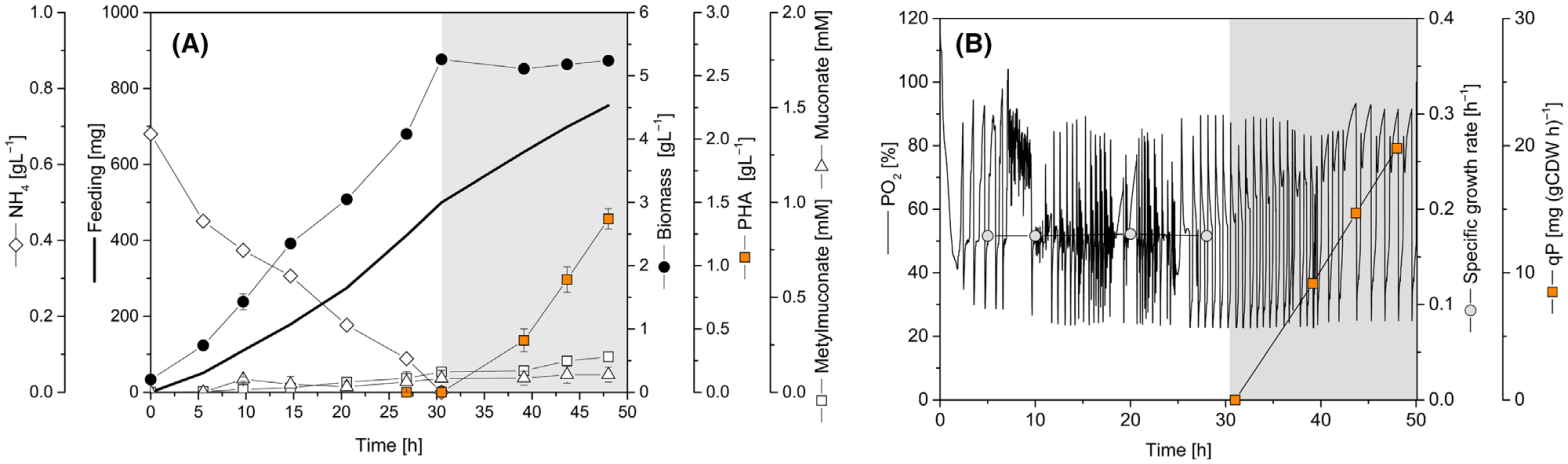
Fed‐batch PHA production using an enriched lignin hydrolysate. A. Substrate and product profile of strain H‐Δ*catA*2 growing on lignin‐derived aromatics. B. DO‐based feed profile, constant specific growth rate and specific PHA productivity. The obtained lignin hydrolysate contained 50 mM of catechol and traces of other aromatics, and it was enriched with 200 mM pure catechol. Grey panels indicate nitrogen limitation. Values represent the mean and errors of two independent bioreactors.

Finally, the PHA producer *P. putida* H‐Δ*catA2* was challenged to produce the biopolymer from a lignin hydrolysate (50 mM catechol) solution and smaller levels of methylated and ethylated catechol derivatives and enriched with 200 mM catechol. The feed addition was coupled to the oxygen demand (DO‐stat) to avoid cell intoxication. A feed pulse (to attain 1.25 mM catechol) was supplied, when the pO_2_ level sharply increased to above 80% of saturation, which indicated a stop of catechol‐dioxygenase catalysis due to catechol depletion (Fig. 8B). The mutant achieved a biomass level of 5.36 g_CDW_ l^–1^ after 30 h, without accumulation of *cis,cis*‐muconate and catechol. Growth stopped when nitrogen became the limiting nutrient. Finally, 1.40 g l^–1^ of PHA was produced, accounting for more than 25% of cell dry weight. The process yielded a final volumetric productivity of 0.7 (g PHA) l^–1^ day^–1^. We recorded a minor accumulation of methyl‐muconate (<200 µM) and traces of ethyl‐muconate (<10 µM) during the PHA production phase, explaining the intrinsic acidification of the medium (Fig 8A).

## Discussion

### The mutant *P. putida* H‐ *Δ*catA2 sets a next level in PHA production from aromatics

One of the biggest challenges to utilizing aromatics for the synthesis of microbial added‐value chemicals is to achieve efficient catabolic conversion without substrate accumulation and/or toxic intermediate’s secretion. In this regard, *P. putida* KT2440, that exclusively exhibits the *ortho*‐cleavage pathway to degrade benzoate and catechol, has been used as both a model strain to investigate the underlying molecular mechanisms on aromatics (Reva et al., 2006; Johnson *et al*., 2017) as well as a biocatalyst for the synthesis of PHA and *cis,cis*‐muconic acid (Linger *et al*., 2014; Kohlstedt *et al*., 2018). So far, most of the PHA production processes on aromatics have been carried out in batch cultures, using low concentrations of benzoate and lignin derivatives since higher levels of the aromatic substrates promote catechol formation arresting biomass and biopolymer synthesis (Linger *et al*., 2014)(Xu *et al*., 2019). The *ortho*‐pathway attenuated mutant *P. putida* H‐Δ*catA2* converted aromatics simultaneously via the *ortho* and *meta* route, yielded much higher PHA levels and accumulated the biopolymer in a fed‐batch process on benzoate with a titre of 6.1 g l^–1^ and a volumetric productivity of 1.8 g l^–1^ day^–1^, surpassing previous efforts approximately six‐fold (Liu et al., 2017). Moreover, 1.4 g l^–1^ PHA was formed from softwood lignin hydrolysate in a cascaded process. The lignin‐derived aromatics served as sole carbon substrate for rapid growth and efficient PHA production, providing an important next step towards future PHA production from lignin (Table 4).

In a recent study, the implementation of the *meta* pathway from a *Sphingomonas* sp. strain into KT2440 along with inactivation of the pyruvate dehydrogenase complex enabled the production of lactate from aromatics (Johnson and Beckham, 2015). In this regard, the balancing of different catabolic routes at the catechol node – i.e. 2,3 *meta*, 4,5 *meta*, and *ortho*‐cleavage pathways – by combining them in one strain (Johnson and Beckham, 2015) and fine‐tuning their activity (this study) emerges as an interesting concept to streamline bio‐based production from aromatics. Similar strategies might also help to drive the production of other important chemicals such as organic acids (Johnson and Beckham, 2015), lipids (Shields‐Menard *et al*., 2017), *cis,cis*‐muconic acid (Barton *et al*., 2018; Becker *et al*., 2018) and adipic acid (Kohlstedt *et al*., 2018; van Duuren *et al*., 2020).

### Simultaneous activation of the ortho and the meta route is the key to achieve high PHA levels from aromatics

Several studies reported that strains of *P. putida,* endowed with the *ortho* and the *meta* route, modulate the activity of the corresponding *ortho* and the *meta* cleaving catechol dioxygenases, C12DO and C23DO, respectively (Kiesel and Müller, 2002), involving a fine‐tuned response to the nutrient level (Barnsley, 1976; Bin and Kai‐Chee, 2008). At a low level of benzoate (< 2 mM), *P. putida* exclusively uses the *ortho* route, and with increasing concentrations, C23DO is activated up to 14‐fold higher activity than C12DO (Hamzah and Al‐Baharna, 1994; Kiesel and Müller, 2002; Bin and Kai‐Chee, 2008). Given the benzoate concentration (15 mM) employed in this study to promote PHA synthesis, the wild‐type strain displayed a C12DO:C23DO ratio of ˜ 9:1, explaining the high secretion of *cis,cis*‐muconate (Fig. 5, Table 3) and the low level of synthesized *mcl*‐PHA. Interestingly, we encountered a different scenario for the mutant strains, where the inactivation of *catA2* was the key to improved PHA formation in *P. putida* H. The deletion had several effects, including the diminished capacity of the *ortho* route at the catechol node by 25% and an increased bioconversion capacity of the *meta* route by almost 8‐fold (Fig. 5). As a result, the total catabolic capability to degrade catechol in *P. putida* H‐Δ*catA2* was even 30% higher than in the wild type. This increased overall flux potential appeared as a surprising response to eliminating an important pathway enzyme on a first glance, although it was highly beneficial for PHA production. As shown, diverting carbon flux into the *meta* pathway in H‐Δ*catA2* increased the availability of acetyl‐CoA and malonyl‐CoA (Fig. 4), which seemed a major driver for enhanced PHA formation. This improvement occurred at the expense of transiently accumulating *cis,cis*‐muconate during the bioconversion (Figs 3 and 7B), which appeared, however, not too severe due to later re‐use by the cells. The growth of all strains without secretion of the intermediate 2‐HMS distinguishes *P. putida* H from other pseudomonads endowed with the *meta* pathway (Bin and Kai‐Chee, 2008; Jiménez et al., 2014). This trait might be a first indication that *P. putida* H channels catechol more efficiently through the *meta* route, resulting in improved precursor supply for PHA formation (Poblete‐Castro *et al*., 2017). Another distinction among the engineered *P. putida* strain was the variation in the monomer composition of the synthesized *mcl*‐PHA (Table 2). Given the catechol accumulation during benzoate bioconversion, this toxic aromatic might trigger changes in acyl‐ACP (C6‐C18) intermediates belonging to the synthesis *de novo* fatty acids to safeguard membrane integrity. Usually, the incorporation of long‐chain unsaturated fatty acids and the increase in the isomerization form, from *cis* to *trans*, are adaptive bacterial mechanisms to cope with catechol and other highly toxic aromatics (Heipieper *et al*., 1992; Mrozik *et al*., 2005). As the *de novo* fatty acid biosynthesis route generates the PHA precursors (3‐hydroxyacyl‐ACP) in *P. putida* strains when grown on aromatics, this might explain to some extent the observed increment on unsaturated monomers in the PHA polymeric chain (Table 2).

Recent studies of benzoate‐grown *P. putida* KT2440 proposed CatA2 as a regulatory safety valve during aromatics degradation, involved in keeping intracellular catechol levels low and avoiding intoxication and cell death (Jiménez et al., 2014). The strain H‐Δ*catA2* did not possess this safety valve anymore so that the upregulation of C23DO in the *meta* branch could display a second or alternative level of safety control. As elucidated from the enzyme assays and flux balance analyses, C23DO has a limited capacity and is negatively controlled by CatA2 (Fig. 4A). It is interesting to note that *P. putida* mt‐2 a bacterium that carries the TOL plasmid pWWO and is endowed with both *ortho* and *meta* routes faced an metabolic conflict for the dissimilation of catechol due to stochastic population heterogeneity as a portion of the cells population remained inactive (Silva‐Rocha and Lorenzo, 2014), which would be interesting to be studied further also in strain H and its mutants.

### Future strategies for metabolic engineering of PHA overproduction in *P. putida* from aromatics

Up to date, the focus for enhanced PHA production in *P. putida* has been poured mainly into engineering pathways involved in the catabolism of fatty acids (Chen and Jiang, 2018; Borrero‐de Acuña *et al*., 2019) and carbohydrates (Poblete‐Castro *et al*., 2013; Borrero‐de Acuña *et al*., 2014; Poblete‐Castro, Binger, *et al*., 2014). Recently, comprehensive metabolic engineering created a *P. putida* PHA factory for biopolymer production from aromatics (Salvachúa *et al*., 2020), which integrated PHA biosynthetic engineering at the level of the *phaZ, PhaG*, PhaC1 and PhaC2 with the elimination of β‐oxidation pathway.

Our results nicely complement these strategies by adding the peripheral catabolism. It seems promising to combine the optimized modules to breed a hyper‐PHA producer with a completely streamlined carbon core metabolism. In addition, and despite its presently weaker performance, further upgrading of engineer the C12DO‐free double deletion strain H‐Δ*catA*Δ*A*2 strain towards deregulation of C23DO activity might be a vital strategy to follow in order to circumvent the accumulation of catechol and carbon loss into muconate. Approaches could involve enhancing PhlH activity using refined synthetic promoters (Zobel et al., 2015; Kohlstedt et al., 2018) to drain as much carbon as possible into the *meta*‐cleavage pathway. In addition, protein engineering of PhlH for improved activity appears promising (Han *et al*., 2015; Becker *et al*., 2018).

Finally, our data suggest that carbon core metabolism might be improved further. The carbon flux distributions revealed significant alterations at the isocitrate and the malate node among the strains. Particularly, the carbon flux into the acetyl‐CoA pool was drastically enhanced in the double mutant, resulting in full activation of the glyoxylate shunt (Fig 6D, Fig. 5B) with isocitrate lyase (ICL) catalysing the first enzymatic step. Generally, activation of the glyoxylate shunt occurs due to a high abundance of pyruvate and acetyl‐CoA (Kukurugya *et al*., 2019). The glyoxylate shunt competes with PHA synthesis for acetyl‐CoA (Klinke *et al*., 2000). Here, we showed increased levels of CoA esters. Hence, the glyoxylate shunt’s inactivation could lead to an improved PHA accumulation from aromatics channelled through the *meta* route exclusively.

## Conclusions

Overall, the *mcl*‐PHA production capacity of the metabolically engineered H‐Δ*catA*2 strain yielded twice as much biopolymer as that produced in the wild‐type strain under various fermentation modes and surpassed previous efforts to derive this important biopolymer from aromatics. By deleting the *catA*2 gene in *P. putida* H, the cells displayed a more balanced operation of the *meta* and *ortho*‐pathways, thus allowing an efficient conversion of the toxic intermediate catechol and generating an enhanced acetyl‐CoA and malonyl‐CoA flux towards PHA synthesis. We further demonstrated that a high cell density culture on benzoate is feasible in *P. putida* H by applying an exponential feeding approach to convert benzoate into biomass and CO_2_ without by‐product formation. Within the PHA production phase under nitrogen limitation, we found different demands for benzoate uptake between the wild‐type and H‐Δ*catA*2 mutant, pointing to the requirement of tailoring the substrate feeding to exploit strain performance fully. Finally, we developed a successful process using lignin hydrolysates from softwood as sole feedstock for fed‐batch PHA synthesis by coupling the oxygen demand (DO‐stat) exerted by the action of catechol dioxygenases during catechol cleavage to prevent substrate overload and growth arrestment along with the full conversion of aromatics into central carbon intermediates for biopolymer synthesis.

## Experimental procedures

### Bacterial strains, plasmids and genetic engineering

All bacterial strains, plasmids and oligos used in this study are summarized in Table 5. The deletion of the genes *catA* (RS04870) and *catA2* (RS18210) from the genome of *P. putida* H used scarless mutagenesis, which relied on DNA excision by *I‐SceI* homing endonuclease (Martínez‐García and de Lorenzo, 2011). In a first step, the oligo pairs catAUpFw‐catADwRv and catA2UpFw‐catA2DwRv were used to amplify the flanking regions of *catA* and *catA*2. Overlap extension PCR was then conducted to fuse the generated up‐ and downstream regions of each gene together, followed by ligation of the joint fragment into the blunt‐end vector pJET1.2. The *E. coli* DH5α strain was used to propagate the obtained pJET1.2 constructs. Afterwards, inserts previously introduced in pJET1.2 vectors were EcoR*I*‐BamH*I* digested and re‐ligated into the suicide vector pSEVA212, which yielded the plasmids pSEVA212‐*catA*UPDW and pSEVA212‐*catA2*UPDW. These were introduced into *E. coli* DH5αλpir. Triparental mating was used to transfer the suicide vectors into *P. putida* H using *E. coli* HB101/pRK600 as a helper strain. The correct integration was validated using PCR with primers catAUpFw/catADwRv and catA2UpFw/catA2DwRv respectively. The gentamicin‐resistance containing plasmid pSEVA628 bearing the *I‐SceI* endonuclease was introduced into the given strains by triparental mating as specified above. Next, 5 mM m‐toluic acid was added to induce *I‐SceI* enzyme expression, which resulted in the formation of kanamycin‐sensitive clones. Successful deletion of *catA* and *catA*2 was controlled via PCR, using primers catAKOFw‐catAKORv and catA2KOFw‐catA2KORv respectively. All deletions were additionally verified by sequencing. The resulting deletion strains were designated *P. putida* H Δ*catA* and *P. putida* H Δ*catA*2. The double deletion strain (*P. putida* H Δ*catA*Δ*A*2) was constructed likewise, transferring the pSEVA212‐*catA*2 vector into the Δ*catA* mutant. All pSEVA628 plasmids were cured by growing strains on Luria Bertani (LB) antibiotic‐free medium three consecutive times. When needed, antibiotics were supplied as follows: 50 μg ml^–1^ kanamycin, 30 μg ml^–1^ chloramphenicol, 150 μg ml^–1^ ampicillin and 15 μg ml^–1^ gentamycin for *E. coli* strains; 500 μg ml^–1^ kanamycin and 50 μg ml^–1^ gentamycin for strains of *P. putida* H.

**Table 5:** Bacterial strains, plasmids and oligos employed in this study

Strains and plasmids	Relevant features	Source/References
*Pseudomonas putida*		
H	Wild‐type strain with a bifurcated aromatic degradation pathway	(Herrmann *et al*., 1987)
H‐*catA*	*P. putida* H‐*catA* knockout mutant strain	This study
H‐*catA2*	*P. putida* H‐*catA2* knockout mutant strain	This study
H‐**Δ** *catA*ΔA*2*	*P. putida* H double knockout mutant strain deficient in the expression of *catA* and *catA2*	This study
*Escherichia coli*		
DH5α	F– Φ80*lacZ*ΔM15 Δ(*lacZYA*‐*arg*F) U169 *rec*A1 *end*A1 *hsd*R17 (rK–, mK+) *pho*A *sup*E44 λ– *thi*‐1 *gyr*A96 *rel*A1	ThermoFisher, Scientific, Darmstadt, Germany
DH5αλpir	*sup* E44, Δ*lacU169* (Φ*lacZ*ΔM15), *recA1*, *endA1*, *hsdR17*, *thi*‐1, *gyrA96*, *relA1*, λpir phage lysogen	Biomedal, Seville, Spain
HB101	Helper strain; F– λ– *hsdS20*(rB– mB–) *recA13 leuB6*(Am) *araC14* Δ(gpt‐ proA)62 *lacY1 galK2*(Oc) *xyl*‐5 *mtl*‐1 *thiE1 rpsL20* (Sm^R^) *glnX44*(AS)	(Benedetti *et al*., 2016)
Plasmids		
pJET1.2	Ap^R^; *oriV* (pMB1) Plasmid suitable for subcloning steps	ThermoFisher, Scientific, Darmstadt, Germany
pRK600	Cm^R^; *oriV* (ColE1), *tra* ^+^ *mob* ^+^ functions from plasmid RK2	(Benedetti *et al*., 2016)
pSEVA212	Km^R^; *oriV* (R6K), Sce‐I RS. Suicide vector	(Martínez‐García *et al*., 2015)
pSEVA628	Gm^R^; *oriV* (RK2), *xylS*‐Pm –> SceI	(Martínez‐García *et al*., 2015)
pJET1.2‐*catA*UPDW	Ap^R^; pJET1.2 plasmid harbouring the fused up‐ and downstream regions of *catA* gene	This study
pJET1.2‐*catA2*UPDW	Ap^R^; pJET1.2 plasmid harbouring the fused up‐ and downstream regions of *catA2* gene	This study
pSEVA212‐*catA*UPDW	Km^R^; pSEVA212 harbouring the fused up‐ and downstream regions of *catA* gene	This study
pSEVA212‐*catA2*UPDW	Km^R^; pSEVA212 harbouring the fused up‐ and downstream regions of *catA2* gene	This study

Cm^R^, chloramphenicol resistance; Km^R^, kanamycin resistance; Gm^R^, gentamycin resistance; Ap^R^, ampicillin resistance; I‐SceI, SceI endonuclease gene precursor; *xylS*‐Pm, genetic circuit inducible upon 3MB addition.

### Media and growth conditions in shake flasks

Cells were plated onto LB agar plates and incubated for 24 h at 30°C. To assess growth profiles, a single colony from an LB plate was inoculated into a 50 ml flask with 10 ml M9 salt minimal medium, which contained 3 g l^–1^ Na_2_HPO_4_·7H_2_O, 12.8 g l^–1^ KH_2_PO4, 0.5 g l^–1^ NaCl, 1 g l^–1^ NH_4_Cl, 0.12 g l^–1^ MgSO_4_·7H_2_O and 1 ml l^–1^ trace element solution (2.7 g l^–1^ CaCO_3,_ 6 g l^–1^ FeSO_4_·7H_2_O, 1.16 g l^–1^ MnSO_4_·H_2_O, 2.0 g l^–1^ ZnSO_4_·H2O, 0.33 g l^–1^ CuSO_4_·5H_2_O, 0.08 g l^–1^ H_3_BO_3_, 0.37 g l^–1^ CoSO_4_·7H_2_O). Sodium benzoate (5 mM) was added as sole carbon and energy source. Strains were grown at 30°C in a rotary shaker at 160 rpm (Ecotron; INFORS HT) using 500 mll baffled shake flasks with 100 ml medium. All cultures were started with an initial optical density OD_600_ of 0.05. For PHA synthesis, cultures were carried out in 500 ml shake flasks with 100 ml modified M9 medium, which contained 15 mM sodium benzoate and 0.2 g l^–1^ NH_4_Cl to trigger N limitation during the process.

### Batch cultures in bioreactors

Cells were grown at 30°C using 1 l (0.8 l working volume) bioreactors (Labfors 5; INFORS HT, Switzerland). For PHA production, the modified M9 medium contained 15 mM sodium benzoate and 0.2 g l^–1^ NH_4_Cl. The aeration rate was set to 0.8 l min^–1^ (1 vvm), using a mass flow controller. The dissolved oxygen (DO) level was maintained at 20% of saturation by controlling the agitation speed up to a maximum of 900 rpm. The pH was automatically controlled at 6.9 ± 0.1 using 1 M NaOH and 1 M H_2_SO_4_.

### Chemostat cultures in bioreactors

Continuous cultivation was carried out under aerobic conditions at a dilution rate of 0.09 h^–1^ using 1 l bioreactors (0.8 l working volume) (Labfors 5l INFORS HT, Switzerland) at 30°C and 900 rpm. The feed solution consisted of modified M9 minimal medium, supplemented with 15 mM sodium benzoate and 0.2 g l^–1^ NH_4_Cl. The pH value was controlled at 6.9 ± 0.1 by automatic addition of 1 M NaOH. The aeration rate was set to 1 l min^–1^ (1.25 vvm). The working volume was kept constant by removing culture broth through a peristaltic pump and controlling the weight with a balance placed under the reactor. Carbon dioxide formation and oxygen consumption was monitored online using a gas analyser (BlueInOne Cell; BlueSens gas sensor GmbH, Herten, Germany) coupled to the condenser outlet of the bioreactor.

### Fed‐batch PHA production on benzoate

By taking an overnight‐grown cell suspension (LB medium), cells were transferred into a 1 l shake flask filled with 300 ml M9 minimal medium (containing 5 mM sodium benzoate and 1 g l^–1^ NH_4_Cl) and cultured in a rotary shaker for 24 h at 160 rpm (Ecotron; INFORS HT). The culture was then used as seed for the fed‐batch process which was operated in a Labfors 5 system (INFORS HT, Switzerland) equipped with a 6‐l vessel. The starting volume was 2 l in all experiments. The temperature was set to 30 °C and the pH was kept constant at 6.9 ± 0.1 by automatic addition of H_3_PO_4_ 4% (w/v) and NaOH 10% (w/v). A modified M9 minimal medium was used for the batch phase, which contained 15 mM sodium benzoate, 1 g l^–1^ NH_4_Cl, and an elevated amount of trace elements (5 ml l^–1^ of the stock given above. The feed solution contained per litre: 3 M sodium benzoate, 12 g MgSO_4∙_7H_2_O and 0.5 g TEGO antifoam (Evonik, Essen, Germany). The airflow was set to 3 l min^–1^ (mixture of air and pure oxygen at a ratio of 5:1). The agitation speed was automatically adjusted in the range of 300–1200 rpm to set the DO level at 20% of saturation. A gas analyser (BlueInOne Cell; BlueSens gas sensor GmbH) was coupled to the exhaust gas of the bioreactor to record CO_2_ production and O_2_ consumption during the process. An exponential feeding strategy was applied during the biomass production phase, following an exponential function (Eq. 1).

(1) $$F\left(t\right)=\mu_{\text{set}}\cdot V_{0}\cdot e^{\left(\mu_{\text{set}}\cdot t\right)}\cdot X_{0}\cdot {\left(S_{0}\cdot Y_{\mathrm{xs}}\right)}^{‐1}$$

Hereby, *F* was the feed rate (l h^–1^), *μ*_set_ the desired specific growth rate (set to 0.15 h^–1^), *S*_0_ the substrate concentration of the feed medium (3 M sodium benzoate), *t* the time after feed start (h), *Y_xs_* the biomass yield on sodium benzoate (taken from Table 1), *V*_0_ the initial volume of the culture (l) and *X*_0_ the initial biomass level (g cells l^–1^). A constant feed was employed during the following PHA accumulation phase, as given below.

### Hydrothermal conversion of lignin

Kraft lignin (Indulin AT, Sigma‐Aldrich) was hydrolysed under supercritical conditions including a subsequent concentration step, as described previously (Kohlstedt *et al*., 2018; Barton*et al*., 2018; van Duuren*et al*., 2020). The lignin concentrate contained a mixture of different aromatics with catechol (50 mM) as major (> 90%) compound and smaller levels of phenol, methylated and ethylated catechol.

### Fed‐batch PHA production on Kraft lignin hydrolysate

For the synthesis of PHA from lignin, a fed‐batch process was carried out in a 100‐ml bioreactor (DASGIP, Jülich, Germany) at 30°C. The batch medium in the bioreactor was modified M9 minimal medium which contained 2 g l^–1^ NH_4_Cl and concentrated lignin hydrolysate (1.25 mM catechol). The feed was composed of concentrated Kraft lignin hydrolysate, which was enriched with catechol to a final concentration of 250 mM so that the fed‐batch process could be maintained for a longer time. The feed additionally contained 0.1 mM sodium benzoate, known as inducer of aromatic catabolism in *P. putida* (Kohlstedt *et al*., 2018). The feed was added to the bioreactor in a pulse‐wise fashion throughout the entire process. The DO level was kept above 20% saturation by suppling filtered air at a rate of 6 l h^–1^ and adjusting the stirrer speed between 200 and 1000 rpm. The pH value was kept constant at 6.9 ± 0.1 by automatic addition of 1.5 M NaOH. Pre‐cultures of strain H‐Δ*catA2* were grown overnight in shake flasks at 30°C and 180 rpm, using 5 mM sodium benzoate plus 2 mM catechol in minimal M9 medium. The process was inoculated to an initial OD_600_ of 0.3. Once catechol was consumed, the DO signal raised to a value above 80%. Then, a pulse was added to replenish the catechol level to 1.25 mM. This feed strategy was repeated through the entire fermentation process.

### Biomass quantification

Cell growth was determined as optical density (OD_600_) using a spectrophotometer (UV‐Vis Optizen 3220UV, Daejeon, Republic of Korea). The cell dry weight (CDW) was determined gravimetrically after harvesting cells from 10 ml culture broth (10 min, 4°C, 9000 *g*) in pre‐weighed tubes, including a washing step with distilled water. The obtained cell pellet was dried at 100°C to constant weight.

### Substrate and product quantification

Benzoate and pathway intermediates (catechol, *cis,cis*‐muconate, 2‐hydroxymuconic semialdehyde), were quantified by LC‐MS, using an HPLC (Eksigent ekspert system; AB Sciex Instruments, MA, USA) coupled to a triple quadrupole mass spectrometer (QTrap 4500; AB Sciex Instruments). Chromatographic separation was based on a mixture of solvent A (aqueous solution of 0.1% of formic acid) and solvent B (acetonitrile 100%) and a RP‐18 column (LiChropher100 Merck, Darmstadt, Germany), set to 40°C at a constant flow rate of 0.8 ml min^–1^. The column was eluted with a linear gradient of (80% A) 0–6 min, (20% A) 6‐7 min and (80% A) 7–10 min. Electrospray ionization was performed in negative mode. The most abundant fragment ions were chosen for selected reaction monitoring, and data were processed using the MultiQuant software. The quantification involved external standards. Ammonium was quantified using a photometric test (Spectroquant Ammonium Test; Merck Millipore, Darmstadt, Germany).

### PHA characterization and quantification

Intracellular PHA was characterized and quantified after collecting 10 ml of culture broth (9000 *g*, 4°C, 10 min), freezing the cell pellet at −20°C for 24 h and freeze‐drying (Lyofilizer VirTis; SP Scientific, New York, NY, USA). The obtained lyophilized cell dry mass (5–10 mg) was subjected to methanolysis, involving incubation in a mixture of 2 ml chloroform, 2 ml methanol, 15% (v v^–1^), concentrated H_2_SO_4_ and 0.5 mg ml^–1^ 3‐methylbenzoic acid for 4 h at 100°C. After cooling down the obtained lysate to room temperature, 1 ml deionized water was added. The mixture was vigorously mixed for 1 min and centrifuged (10 min, 7000 *g,* 20°C). The bottom phase, containing methyl esters of the PHA monomers, was collected and analysed using GC/MS (YL6900; Young Instruments, Anyang, Korea) (Borrero‐de Acuña *et al*., 2017), including calibration with purified PHB (Sigma‐Aldrich, MI, USA) and purified *mcl*‐PHA from previous work (Oliva‐Arancibia *et al*., 2017). The individual monomers were identified by their mass spectrum (NIST 17 Mass Spectral Library, MA, USA). The relative fraction of PHA in the cell (%) was defined as the amount (grams) of the biopolymer divided by the total cell dry mass (grams), multiplied by 100.

### Enzymatic assays

Cells were harvested (40 ml), centrifuged (8000 *g*, 5 min, 4°C) and washed with 20 ml Tris–HCl buffer (TB) (50 mM, pH 8.0). The obtained cell pellet was resuspended in 1 ml TB. Cells were then disrupted by sonication (QSonica, Newtown, CT, USA) with 30‐sec intervals on ice in between (three cycles, 30 s). DNase I (1 mg ml^–1^) and RNase (0.25 mg ml^–1^) were added in between. Cell debris was removed by centrifugation (13 000 *g,* 4°C, 1 h). The obtained cell extract was immediately used for enzymatic analysis. The protein concentration in cell extract was quantified by the Bradford method using bovine serum albumin as the standard. For assaying the activity of catechol 1,2‐dioxygenase (E.C. 1.13.11.1) and catechol 2,3‐dioxygenase (E.C. 1.13.11.2), the reaction mixture (1 ml) contained 100 μl cell extract, 50 mM Tris‐HCl (pH 8.0) and 20 μM catechol. The enzymatic conversion of catechol into *cis,cis*‐muconate and 2‐hydroxymuconic semialdehyde (2‐HMS) was followed by measuring the change in absorbance at 30°C at 260 nm (ɛ = 16 800 M^−1^ cm^−1^) and 375 nm (ɛ = 46000 M^−1^ cm^−1^) respectively. Malic enzyme (MAE) activity (E.C. 1.1.1.40) was assayed at 30°C by monitoring the oxidative decarboxylation of l‐malic acid in a reaction mixture (3 ml) containing 300 µL cell extract, 67 mM Tris–HCl (pH 7.4), 3.3 mM l‐malic acid, 0.3 mM *β*‐nicotinamide adenine dinucleotide phosphate and 5.0 mM MnCl_2_. The reaction was monitored by recording the change in absorbance at 340 nm (ɛ = 62200 M^−1^ cm^−1^). Isocitrate lyase (ICL) activity (E.C. 4.1.3.1) was measured at 30°C in a 1 ml reaction mixture containing 30 mM imidazole (pH 6.8), 5 mM MgCl_2_, 1 mM EDTA, 4 mM phenylhydrazine, 1 mM *DL*‐isocitric acid and 100 µl cell extract. The formed glyoxylate‐phenylhydrazone was monitored spectrophotometrically at 324 nm (ɛ = 3200 M^−1^ cm^−1^). For all assays, one unit of enzyme activity (U) was defined as the amount of enzyme necessary to form 1 µmol product per min. Specific enzyme activity was defined as unit (U) per mg of protein.

### Quantification of intracellular CoA thioesters

Sampling, sample processing and analysis of intracellular CoA thioesters were conducted as described recently for the related strain *P. putida* KT2440, involving simultaneous quenching and extraction (95% acetonitrile, 25 mM formic acid, −20 °C), sample clean up, CoA ester analysis by a triple quadrupole MS (QTRAP 6500+; AB Sciex, Darmstadt, Germany) coupled to an HPLC system (Agilent Infinity 1290 System; Agilent Waldbronn, Waldbronn, Germany) and absolute quantification against an internal ^13^C enriched standard (Gläser *et al*., 2020).

### Flux balance and robustness analysis

Flux balance analysis was used to predict growth and carbon flux patterns during PHA‐producing conditions (Beckers *et al*., 2016). A draft metabolic model of the *P. putida* H strain was created based on the genome sequence of the wild type (Vizoso *et al*., 2015). Flux balance analysis used the COBRA Toolbox v2.0 (Schellenberger *et al*., 2011) within the MATLAB environment (The MathWorks Inc., MA, USA), using Gurobi Optimization (Gurobi GmbH, Berlin, Germany) for solving the linear programming problem. The constraint‐based model comprised 70 reactions, of which 16 reversible conversions in 8 exchange reactions (Supporting information). The precursor demand for biomass formation was taken from previous work (Poblete‐Castro *et al*., 2013). As usual in stoichiometric modelling, the reaction network was balanced regarding elemental composition, charge and degree of reduction (Roels, 1980).

### Statistical analysis

All shake flask experiments were conducted as three biological replicates and average values were shown with deviation of the repetition as error bars. Experiments in bioreactors were carried out in duplicates. Using GraphPad Prism 5.0 (GraphPad Software, Inc., San Diego, CA, USA), we compared the resulting values of the groups via one‐way analysis of variance (ANOVA). Differences between groups were considered significant at a *P* value of < 0.05.

## Conflict of interest

None declared.

## Supporting information


**Fig. S1**. Robustness analysis of the catechol branch of *P. putida* H with a benzoate uptake rate set at 2.4 [mmol (gCDW×h)^‐1^].
**Table S1**. Oligos employed in this work.
**Table S2**. *In‐silico* model of *Pseudomonas putida* H.
