# Supplementary material for: Channelling carbon flux through the meta‐cleavage route for improved poly(3‐hydroxyalkanoate) production from benzoate and lignin‐based aromatics in Pseudomonas putida H
Source: Microb Biotechnol. 2020 Nov 10;14(6):2385–402. doi: 10.1111/1751-7915.13705 (PMC8601166; doi:10.1111/1751-7915.13705)

Production of poly(3-hydroxyalkanoate) from benzoate and lignin-derived aromatic compounds using metabolically engineered *Pseudomonas putida* H

José Manuel Borrero-de Acuña[1#], Izabook Gutierrez-Urrutia[1,2#], Cristian Hidalgo-Dumont[1], Carla Aravena-Carrasco[1], Matias Orellana-Saez[1], Nicolas Pacheco[1], Nestor Palominos-Gonzalez[1], Jozef B.J.H. van Duuren[2], Viktoria Wagner[2], Judith Becker[2], Michael Kohlstedt[2], Flavia C. Zacconi[3,4], Christoph Wittmann[2], Ignacio Poblete-Castro[1*]

Supplementary Material

Table S1. Oligos employed in this work.

Oligos (5´ → 3´)	Sequence	Source
*catA*UpFw	GAATTCGCACCATGCTCGAAGGTT	Fermelo Biotec, Santiago, Chile
*catA*UpRv	CACAGTGACATAACCTCGAACCGTGCTTACCTCGTATTGTTC	Fermelo Biotec, Santiago, Chile
*catA*DwFw	GTTCGAGGTTATGTCACTGTG	Fermelo Biotec, Santiago, Chile
*catA*DwRv	GGATCCTCACCAGGAAGTTGAGCAAG	Fermelo Biotec, Santiago, Chile
*catA*2UpFw	GAATTCGTCTACGCCCTGAGCTCCT	Fermelo Biotec, Santiago, Chile
*catA*2UpRv	AAACAGCCCACTCGGTGGAGGGGGTTACCTCGTCTTGTT	Fermelo Biotec, Santiago, Chile
*catA*2DwFw	TCCACCGAGTGGGCTGTTT	Fermelo Biotec, Santiago, Chile
*catA*2DwRv	GGATCCATGCCAAAGCTGAACAGGAT	Fermelo Biotec, Santiago, Chile
catAKOFw	CCGTGAAAATTTCCCACACT	Fermelo Biotec, Santiago, Chile
catAKORv	GGGTCGAATCGAAGTACGAA	Fermelo Biotec, Santiago, Chile
catA2KOFw	CATACTGCCGAGGTACAGCA	Fermelo Biotec, Santiago, Chile
catA2KORv	GTCCCCCGACAGGTTTATCT	Fermelo Biotec, Santiago, Chile

S2. *In-silico* model of *Pseudomonas putida* H

Pathways	Reactions
Transport Reactions	'→ BEN(e)'
	'→ NH3(c) '
	' → SO4(c) '
	' → O2(c) '
	'biomass(c) → '
	'CAT_ex(e) → '
	'HMS_ex(e) → '
	'MUC_ex(e) → '
	'ATPmaintenance(c) → '
	'CO2(c) → '
Benzoate uptake and conversion to catechol	'BEN(e) → BEN(p)'
	'BEN(p) → BEN(c)'
	'BEN(c) + NADH(c) + O2(c) → BENDIOL(c) + NAD(c)'
	'BENDIOL(c) + NAD(c) → CAT(c) + CO2(c) + NADH(c)'
Ortho pathway	'CAT(c) + O2(c) → MUC(c)'
	'MUC(c) → MUC_ex(e)'
	'MUC(c) MUCLAC(c)'
	'MUCLAC(c) → KAD(c)'
	'KAD(c) + SUCC-CoA(c) → KAD-CoA(c) + SUCC(c)'
	'KAD-CoA(c) → AcCoA(c) + SUCC-CoA(c)'
Meta pathway	'CAT(c) + O2(c) → HMS(c)'
	'HMS(c) + NAD(c) → HM(c) + NADH(c)'
	'HMS(c) → HMS_ex(e)'
	'HMS(c) → FOR(c) + 2KPE(c)'
	'FOR(c) + NAD(c) → CO2(c) + NADH(c)'
	'HM(c) → 2KHE(c)'
	'2KHE(c) → 2KPE(c) + CO2(c)'
	'2KPE(c) → 4HKPE(c)'
	'4HKPE(c) → acetA(c) + PYR(c)'
	'acetA(c) + NAD(c) → AcCoA(c) + NADH(c)'
Pentose phosphate pathway	'RIB-5P(c) ⇔ XYL-5P(c)'
	'RIB-5P(c) ⇔ RIBO-5P(c)'
	'S7P(c) + GAP(c) ⇔ RIBO-5P(c) + XYL-5P(c)'
	'S7P(c) + GAP(c) ⇔ E4P(c) + F6P(c)'
	'F6P(c) + GAP(c) ⇔ E4P(c) + XYL-5P(c)'
Entner-Doudoroff pathway	'6PG(c) → KDPG(c)'
	'KDPG(c) → GAP(c) + PYR(c)'
Embden-Meyerhof-Parnas pathway	'G6P(c) ⇔ F6P(c)'
	'FBP(c) → F6P(c)'
	'FBP(c) ⇔ GAP(c) + DHAP(c)'
	'DHAP(c) ⇔ GAP(c)'
	'GAP(c) + NAD(c) ⇔ 13-PG(c) + NADH(c)'
	'ADP(c) + 13-PG(c) ⇔ ATP(c) + 3-PG(c)'
	'3-PG(c) ⇔ 2-PG(c)'
	'2-PG(c) ⇔ PEP(c)'
	'PEP(c) + ADP(c) → PYR(c) + ATP(c)'
	'PYR(c) + NAD(c) → AcCoA(c) + NADH(c) + CO2(c)'
	'PYR(c) + 2 ATP(c) → 2 ADP(c) + PEP(c)'
TCA cycle	'AcCoA(c) + OAA(c) → CIT(c)'
	'CIT(c) ⇔ ICI(c)'
	'ICI(c) + NADP(c) → AKG(c) + CO2(c) + NADPH(c)'
	'AKG(c) + NAD(c) → SUCC-CoA(c) + NADH(c) + CO2(c)'
	'SUCC-CoA(c) + ADP(c) ⇔ SUCC(c) + ATP(c)'
	'SUCC(c) + Q(c) ⇔ FUM(c) + QH2(c)'
	'FUM(c) → MAL(c)'
	'MAL(c) + NAD(c) ⇔ OAA(c) + NADH(c)'
Glyoxylate metabolism	'ICI(c) → Glyoxy(c) + SUCC(c)'
	'Glyoxy(c) + AcCoA(c) → MAL(c)'
Amphibolic metabolism	'PYR(c)+ ATP(c) + CO2(c) → OAA(c) + ADP(c)'
	'MAL(c) + NADP(c) → PYR(c) + NADPH(c) + CO2(c)'
	'OAA(c) → PYR(c) + CO2(c)'
	'PEP(c) + CO2(c) → OAA(c)'
	'(5) AcCoA(c) + (4) ATP(c) + (7) NADPH(c) → PHA(c) + (4) ADP(c) + (7) NADP(c)'
PHA synthesis	'PHA(c) → '

Energy metabolism	'NADPH(c) + NAD(c) → NADP(c) + NADH(c)'<br>'(3) NADH(c) + (3) NADP(c) + ATP(c) → (3) NAD(c) + (3) NADPH(c) + ADP(c)'<br>'(0.5) O2(c) + NADH(c) + (1.33) ADP(c) → NAD(c) + (1.33) ATP(c)'<br>'(0.5) O2(c) + QH2(c) + (0.66) ADP(c) → Q(c) + (0.66) ATP(c)'<br>'ATP(c) → ADP(c) + ATPmaintenance(c)'<br>'SO4(c) + (3) NADPH(c) + (4) ATP(c) → H2S(c) + (3) NADP(c) + (4) ADP(c)'
Biomass production	'(1.481) OAA(c) + (1.338 3)-PG(c) + (0.627) RIBO-5P(c) + (17.821) ATP(c) + (16.548) NADPH(c) + (6.965) NH3(c) + (3.548) NAD(c) + (2.930) AcCoA(c) + (2.861) PYR(c) + (1.078) AKG(c) + (0.361) E4P(c) + (0.72) PEP(c) + (0.233) H2S(c) + (0.072) F6P(c) + (0.206) G6P(c) + (0.129) GAP(c) → biomass(c) + (16.548) NADP(c) + (3.548) NADH(c) + (17.821) ADP(c) + (1.678) CO2(c)'

S3. Robustness analysis of the catechol branch of *P. putida* H with a benzoate uptake rate set at 2.4 [mmol (gCDW·h)$^{-1}$].

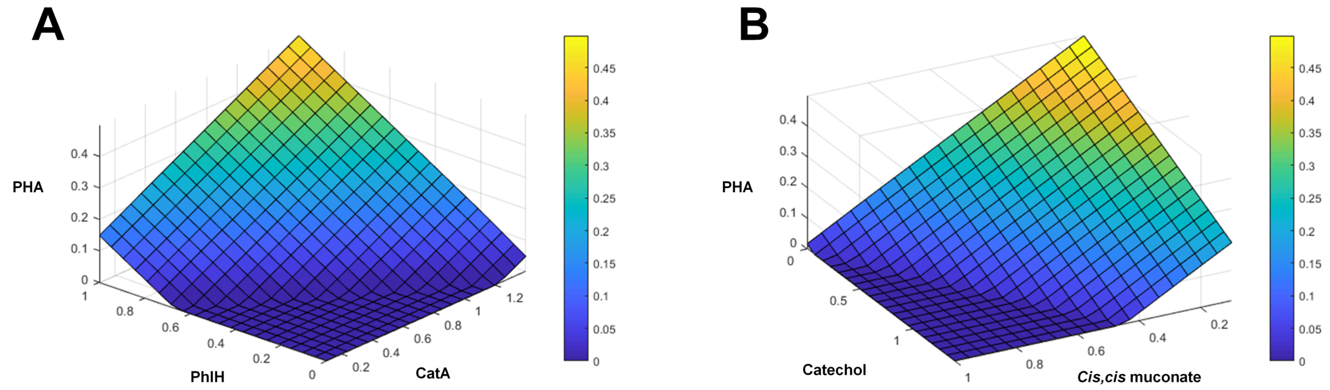

Supplement: Supplementary file 1 — Fig. S1. Robustness analysis of the catechol branch of P. putida H with a benzoate uptake rate set at 2.4 [mmol (gCDW×h)‐1]. Table S1. Oligos employed in this work. Table S2. In‐silico model of Pseudomonas putida H. [file MBT2-14-2385-s001.pdf]
